# Effect of Gamification on Improved Adherence to Inhaled Medications in Chronic Obstructive Pulmonary Disease: Randomized Controlled Trial

**DOI:** 10.2196/65309

**Published:** 2025-05-14

**Authors:** Xiting Huang, Zhili Jiang, Yifan Dai, Yang Liu, Ziying Dai, Jing Wang, Liping Chen, Zhiqian Wang, Wenxiao Wu, Lihua Huang

**Affiliations:** 1 Department of Nursing The First Affiliated Hospital Zhejiang University School of Medicine Hangzhou China; 2 Respiratory Medicine The Affiliated Hospital of Hangzhou Normal University Hangzhou China

**Keywords:** chronic obstructive pulmonary disease, COPD, inhaled medication, medication adherence, gamification, Fogg Behavior Model, randomized controlled trial

## Abstract

**Background:**

Inhaled medication is the preferred route of administration for patients with chronic obstructive pulmonary disease (COPD). The compliance rate of inhaled medication in patients with COPD is <50%, which increases the risk of acute exacerbations. Considering the complex steps of inhaled medication, improving inhaled medication compliance not only requires consistent medication frequency and medical advice but also an evaluation of whether the patient has mastered the inhaler technique to achieve the correct dose.

**Objective:**

This study aimed to explore the effectiveness of an inhaled medication education program (Inhaling-Health website) based on the Fogg Behavior Model and gamification design on inhaled medication compliance and other health-related outcomes in patients with COPD.

**Methods:**

In a randomized, 2-arm, concurrent parallel design, we enrolled 102 patients with COPD from respiratory medicine clinics at 2 hospitals in Zhejiang Province, China, between April and May 2023. Participants were randomly assigned to either the control group (51/102, 50%) or the experimental group (51/102, 50%). All participants completed the intervention, with 94 participants completing 6 months of follow-up. Two independent-sample 2-tailed *t* tests and the Mann-Whitney U test were used to analyze group differences, and generalized estimating equations were used to analyze repeated measurements.

**Results:**

After the intervention, all outcome measures improved compared to baseline. The generalized estimating equation showed that, compared to the control group, the Inhaling-Health website led to a gradual improvement in total adherence-to-inhaler scores starting from 2 months after the intervention (median 51.00, IQR 49.00-52.25 vs median 50.00, IQR 47.00-51.00; *Z*=–2.014; *P*=.04). It had a separate positive effect on inhaler technique. In addition, the website was more effective in reducing the modified Medical Research Council score than routine inhaled medication education, starting from 4 months after intervention (median 1.00, IQR 1.00-2.00 vs median 1.00, IQR 0.00-2.00; *Z*=–2.260; *P*=.02). The website was also more effective than the conventional intervention in improving Chronic Obstructive Pulmonary Disease Knowledge Questionnaire scores, beginning at the end of the intervention (mean 6.14, SD 1.83 vs mean 7.06, SD 1.82; *t*_1_=–2.551; *P*=.01).

**Conclusions:**

The gamified inhaled medication education program based on the Fogg Behavior Model can improve inhaled medication adherence, inhaler technique accuracy, health literacy, lung function, and health-related quality of life; help reduce the severity of dyspnea and COPD physical symptoms; and reduce the risk of acute exacerbations in patients with COPD. This study can provide a reference for inhaled medication education in patients with COPD.

**Trial Registration:**

China Clinical Trials Registry (ChiCTR) ChiCTR2300070213; https://www.chictr.org.cn/showprojEN.html?proj=194829

## Introduction

### Background

Chronic obstructive pulmonary disease (COPD) is a heterogeneous lung disease that ranks among the top 3 causes of death and the top 5 disease burdens worldwide [1-3]. According to guidelines [1,4], inhaled medication is highly recommended for administration as it delivers the drug directly to the lungs and respiratory tract while avoiding hepatic first-pass effects [5,6]. However, the effectiveness of inhalation therapy largely depends on patient compliance with their prescribed inhaled medication, meaning patients with COPD should use their inhalation device as prescribed in time and quantity [7]. Studies have shown that the adherence rate of inhaled medication in patients with COPD ranges from 10% to 50% [3,8], with compliance gradually decreasing within 6 months [9]. Airway obstruction or stenosis, cough, dyspnea, and poor respiratory coordination make it difficult for patients with COPD to use inhalers properly [10]. A systematic evaluation of 144 inhaler technique observation reports indicates that only 31% (95% CI 28%-35%) of patients with COPD demonstrated the best inhaler technique in the past 4 decades [11]. Therefore, poor adherence to inhaled medication increases the rate of acute exacerbation and readmission of patients with COPD, which increases the burden on the medical system and the economic burden of patients [12].

The use of inhalation devices is a complex process that requires guidance from medical professionals to assist patients with COPD in completing various steps, such as opening the device, loading drugs, exhaling residual gas before inhalation, inhaling drugs, holding breath, gargling, and cleaning inhalation devices [13]. However, the extensive educational content may hinder older patients with COPD from effectively mastering the techniques. This raises concerns about whether patients are correctly inhaling their medication over the long term [14,15]. In addition, lengthy and monotonous educational materials may lack appeal for patients with COPD [16]. In clinical practice, medical professionals provide health education manuals [17] and videos demonstrating proper inhaler techniques [18], which result in passive patient education. Without supervision or face-to-face repeated education sessions, it is difficult to determine the effectiveness of these methods. Thus, inhaled medication education needs to use mobile health to provide patients with step-by-step, interactive medication education, enhancing their motivation and self-efficacy. This approach can help patients with COPD develop proper inhaler use habits.

Fogg [19] put forward the formula “B=MAP” to explain the mechanism of human behavior, which means target behavior only occurs when people have high motivation, ability, and some prompt at the same time. Higher motivation increases the likelihood of achieving the target behavior. People are more likely to engage in behaviors that require less time, money, physical or mental effort, and social pressure, especially when those behaviors align with their existing habits. These can indirectly affect a person’s ability to behave. Behavioral prompt refers to the external stimulus for people to complete a behavior [20]. Fogg and Euchner [21] also emphasized the importance of “timely celebration” of influencing habit formation. Therefore, in addition to improving inhaler technique through education, timely feedback and mobilization of positive emotions are also essential to improve adherence to inhaled drugs.

With the development of smartphones and wireless monitoring devices, the collaboration between mobile health and chronic disease management is increasing. Communication, data collection, patient monitoring, and education have facilitated chronic disease management [22]. Gamification, derived from video games, has been shown to enhance motivation for healthy behaviors and maintain a higher level of patient participation in chronic disease management [23]. Currently, game elements and mechanisms have successfully boosted self-management enthusiasm among patients with asthma [24], diabetes [25], cancer [26], coronary heart disease [27], obesity [28], and stroke [29]. Patients can complete symptom management, medication management, exercise rehabilitation, and health education as part of an enjoyable gaming experience [30]. Therefore, gamification is not about entertainment but about creating positive emotions through timely feedback and transforming self-management tasks into spontaneous behavioral habits. Previous studies have found that patients with COPD can use mobile health technology and wearable devices to complete pulmonary rehabilitation through exercise videos and somatosensory games [31,32]. However, it remains unclear whether gamified educational interventions involving inhaled drugs are as effective as exercise.

### This Study

This study aimed to investigate whether gamified education can help improve inhaled medication adherence in patients with COPD and whether its effect can be sustained for 6 months. We hypothesize that participants who receive education on gamified inhalation medication will improve compliance with their inhalation medication and correct inhaler technique following the intervention. In addition, we anticipate improvements in health literacy, reduction in alleviation of dyspnea severity, and enhancement of health-related quality of life (HRQoL) compared to the contemporaneous control group. Furthermore, we also hypothesize that the gamified intervention will have positive effects on the frequency of reduced acute exacerbations and pulmonary function in patients with COPD.

## Methods

### Study Design

Due to the use of convenience sampling, it was challenging to achieve a strictly random sampling design. This study used a randomized design (2-arm, concurrent parallel, pretest-posttest design). Patients with COPD who met the inclusion criteria were selected from the Respiratory Medicine Clinic of First Affiliated Hospital of Zhejiang University School of Medicine (a provincial-level grade-A tertiary general hospital) and Hangzhou Normal University Affiliated Hospital (a grade-A tertiary general hospital of a prefectural-level city in Hangzhou) between April 2023 and May 2023 by convenience sampling. To better control the confounding variables, computer-generated numbers were randomly assigned to represent patients with COPD who met the inclusion criteria in a 1:1 ratio of odd and even numbers. Odd numbers were allocated to the control group, while even numbers were allocated to the experimental group. The group assignments were concealed from both the investigator and the statistical analyst of the research team.

### Ethical Considerations

This study was approved by the institutional research board of the First Affiliated Hospital of Zhejiang University School of Medicine (2022 No. 1088- Quick) and the Hangzhou Normal University Affiliated Hospital (2022 (E2) -HS- 180). The study was also registered on China Clinical Trials Registry (ChiCTR2300070213). All ethical considerations were conducted in accordance with the Helsinki Declaration, and written informed consent was obtained from all participants. We did not include study participant information in this manuscript, and any individual names in the supplementary material have been masked. No compensation was provided to the participants.

### Participants

Participants were recruited from the respiratory medicine clinics in 2 hospitals. The inclusion criteria for the participants were as follows: (1) the diagnostic criteria according to the guidelines of Global Initiative for Chronic Obstructive Lung Disease (GOLD) [1], forced expiratory volume in the first second (FEV1), and forced vital capacity (FVC) <0.70; (2) capability to use communication software WeChat (version 8.0.55; Tencent Computer Systems Co, Ltd; with the help of caregivers); (3) inhalation preparation for budesonide formoterol powder inhalant (Symbicort Turbo), salmeteroticasone powder inhaler (Sulatide), tiotropium bromide powder inhaler (Selivar), tiotropium bromide powder aerosol (Neberol), indatarrolonium bromide inhaler aerosol (Jerun), or budegforo inhalation aerosol (Bezurin); and (4) the ability to provide informed consent. The exclusion criteria were as follows: (1) other respiratory diseases, such as lung cancer, asthma, and pneumonia; (2) serious comorbidities, such as renal insufficiency and cardiac failure; (3) conditions affecting respiratory function, such as chest fracture; (4) diagnosed mental disorders or cognitive dysfunction; (5) inability to use smartphones due to hearing or visual impairments; and (6) participation in other intervention studies before enrollment in this study.

We calculated the sample size based on the assumption that patients were educated on inhalation medication. The ratio of the sample size of the 2 groups was 1:1. The calculation formula for the sample size was n1 = n2 = (Zα + Zβ) 2 × 2σ2 / δ2 (with the 2-sided test, where α=0.05 and β=0.10). The same study design was used to conduct a pilot study with 10 individuals in the experimental group and 10 in the control group (σ=3.72 and δ=3.65 were substituted into the formula). Considering a 20% loss in sample size during the follow-up, we needed to recruit at least 54 people (at least 27 cases in each group). Because of the small sample size of the calculated results, we aimed to recruit as many participants as possible to minimize the risk of false positive results.

### Procedure

Before starting the project, we formed a panel of experts to develop a gamified inhaled medication education website, Inhaling-Health. The experts included 2 physicians with senior professional titles who have worked in the respiratory medicine department for >10 years, 1 nurse with senior professional title who has been engaged in nursing informatics and nursing quality management for >20 years, 3 nurses with associate senior professional titles who have worked in the respiratory ward for >10 years, 1 head nurse who has been engaged in outpatient inhalation medicine education for 5 years, 2 nursing graduate students, 1 computer engineer, and 1 game artist.

The relevant evidence for inhaled medication education and management was collected through evidence-based research [33]. The intervention period for this study was 1 month, and a 6-month follow-up survey was conducted [34]. In addition, because each inhaler device provides a 30-day supply, patients with COPD should visit the hospital regularly for monthly follow-up appointments to receive their medication. The aforementioned conditions laid the foundation for the successful development of this study.

**Figure 1:**
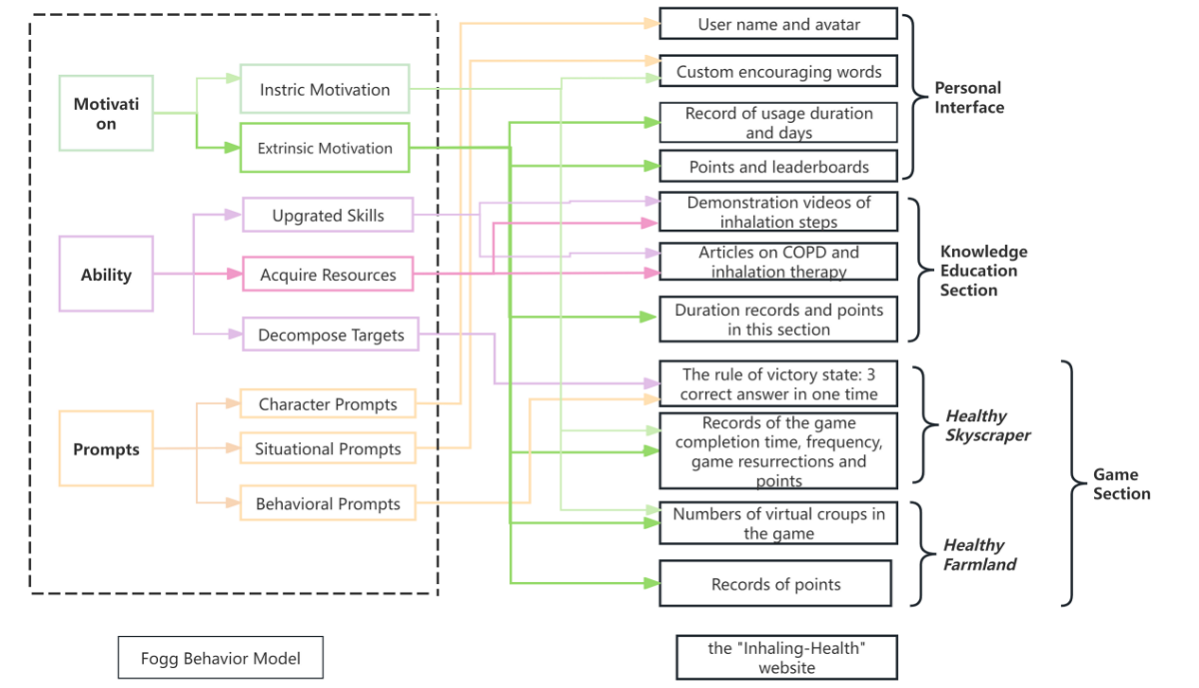
The functional framework of the Inhaling-Health website. COPD: chronic obstructive pulmonary disease.

**Figure 2:**
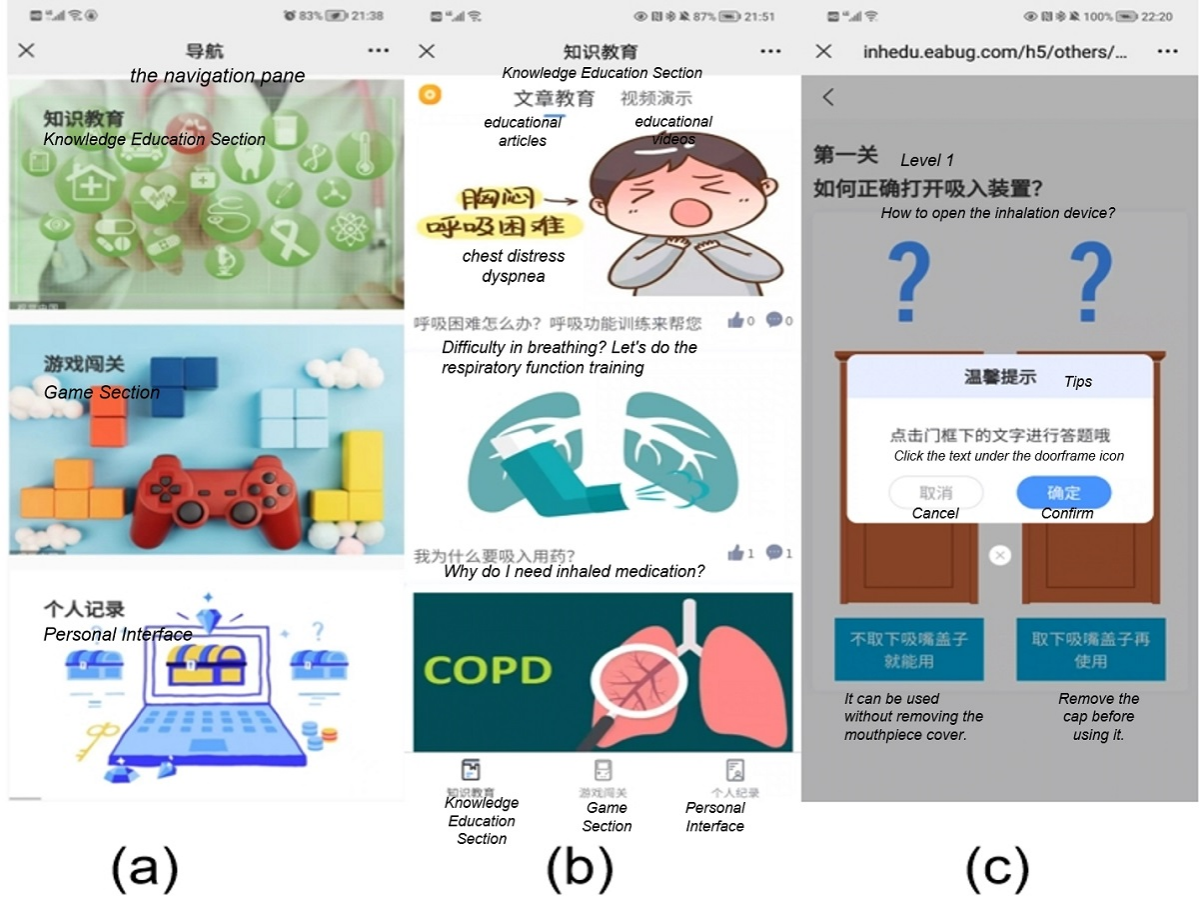
Screenshot of the Inhaling-Health website: (A) the navigation pane of the Inhaling-Health website, (B) the educational articles of the Knowledge Education section, and (C) the start interface of the game Healthy Skyscraper of the Game section.

On the basis of the Fogg Behavior Model [20] and gamification design principles, combined with the evidence-based study methods and panel discussions, we constructed an initial functional framework for the Inhaling-Health website (Figure 1). Figure 2 shows the navigation pane of the website (more details are provided in Multimedia Appendix 1). After the pilot study, qualitative interviews were conducted with participants in the experimental group to identify the user needs and improve the website design.

Patients who came for COPD appointments were approached by a clinical research associate and asked to join the study. If they agreed, they were entered into the study. We recruited participants who met the criteria in the respiratory medicine clinic, and then explained the study purpose and intervention method. Following this, we asked the participants to sign the informed consent. In this study, the same investigator assisted participants in providing personal information and completing the as outcome measures before intervention (T0). The clinical research associate took the participants to 2 independent clinics for inhalation medication education based on their group assignments. On the basis of the evidence from the team’s findings and related studies [9,11], the intervention lasted 1 month, followed by a 6-month follow-up period. To minimize estimation bias, data were collected by the same investigator before the intervention (T0), after the intervention (T1), and monthly during the 6-month follow-up period (T2-T7).

### Intervention

Both the intervention and control groups received routine face-to-face health education guidance, which included basic knowledge of the disease; the importance of inhalation medication; guidance on the use of inhalation devices; the duration of pulmonary function review; and education on self-management skills, such as smoking cessation, breathing training, and nutritional support. In addition to this routine guidance, the intervention group used a self-made gamified inhalation medication education platform called Inhaling-Health website, which was designed based on the Fogg Behavior Model [20]. The researcher responsible for the intervention in the experimental group introduced the participants face-to-face and provided demonstrations to ensure proper use. Participants in the experimental group were required to use the Inhaling-Health website at least once a week. The intervention implementer monitored participant use through user data from the health care site of the website. A reminder message would be sent on the sixth day of each week to urge those who had not used the Inhaling-Health website to maintain the intervention quality.

### Measures

The general data of the participants were evaluated by a self-designed questionnaire (Multimedia Appendix 2). This questionnaire, developed by the researchers after conducting a comprehensive review of relevant literature, comprised three parts: (1) demographic information, such as age and gender; (2) disease-related information, including COPD course, GOLD grade (the assessment of airflow obstruction severity based on the postbronchodilator value of FEV1, from GOLD Ⅰ to GOLD Ⅳ) [1], smoking status, and comorbidities; and (3) inhaled medication details, such as duration of inhalation delivery, type of inhaler, number of inhalers, and inhalation regimen.

The primary outcome measures of this study included compliance with inhaled medication and correctness of inhaler technique. The Test of Adherence to Inhalers (TAI) questionnaire was used to assess compliance with inhaled medication in patients with COPD [35]. This questionnaire was developed by Plaza et al [35], specifically to identify various reasons for poor adherence to inhaled medication in patients with COPD and patients with asthma. Patients completed the first 10 items using a 5-point Likert scale, with scores ranging from 1 for “always” to 5 for “never.” A higher score indicated better compliance with inhaled medication. Items 1 to 5 represented the dimension of the erratic nonadherent behavioral pattern, indicating occasional behavior related to poor compliance, while items 6 to 10 represented dimensions of the deliberate nonadherent behavioral pattern, indicating intentional behavior related to poor compliance. Items 11 to 12 were completed by medical professionals, using a score range of 1 to 2, to identify patients who did not understand the frequency, dosage, or the technique for using their inhaler. The TAI questionnaire had been translated into Chinese and used in the investigation of patients with COPD. The reliability and content validity were acceptable (Cronbach α=0.843; content validity index=0.966) [36]. Due to the complexity of the steps in using inhaled medication, it was challenging to comprehensively evaluate the inhaler technique in patients with COPD based solely on 1 item in the TAI questionnaire. The suction operation questionnaire referred to the 7-step method of British pharmacist Murphy [37], combined with characteristics specific to the use of inhalation devices by Chinese patients with COPD, including a total of 10 steps (sitting straight, opening the device, loading drugs, exhaling, holding the mouthpiece, inhaling, breath holding, repeating, cleaning, and gargling) [38]. Considering differences among different types of inhalation devices, the accuracy of inhaler technique was evaluated by calculating the accuracy rate. The accuracy rate was determined using the following formula: (number of steps used for the correct suction device / the number of suction device steps required for the device) × 100%.

Secondary outcome measures in this study included the severity of dyspnea, HRQoL, health literacy, frequency of acute exacerbation, and pulmonary function. The modified Medical Research Council (mMRC) scale was the tool recommended by the GOLD report to assess the severity of dyspnea in patients with COPD [1]. It was graded from 0 to 4 based on the level of activity when experiencing shortness of breath [39]. The COPD Assessment Test (CAT) was used to assess the severity of the impact of COPD on a patient’s health, with a range of 0 to 40, reflecting the HRQoL [40]. The Chinese version of the CAT had good internal consistency and reliability (Cronbach α=0.805) [41]. Health literacy among patients with COPD was evaluated by the Chinese version of the Chronic Obstructive Pulmonary Disease Knowledge Questionnaire (COPD-Q) [42], including 8 forward knowledge questions and 5 reverse knowledge questions, ranging from 0 to 13. The Cronbach α of the Chinese version of COPD-Q was 0.72. To ensure comparability before and after intervention, the frequency of acute exacerbations in both groups of patients with COPD was assessed over a half-year period. Pulmonary function tests were performed in the hospital’s pulmonary function laboratory, with direct measurements including forced expiratory volume percentage, FEV 1, FVC, and the FEV1/FVC ratio. The number of active days and the frequency of each function of the website were also monitored to achieve the quality of the intervention in the experimental group. The system usability scale (SUS) was used to evaluate user satisfaction with the Inhaling-Health website [43].

The data from T0 were collected in person by the investigator in the outpatient department; the data from T1 to T7 could be collected web-based or offline based on the patient’s dispensing status. During follow-up appointments at the hospital clinic, the investigator would personally guide the patient to complete the TAI, mMRC, CAT, and COPD-Q questionnaires; demonstrate the operation with the patient-prescribed inhalation devices; and score the operation steps of the inhaler technique. For patients choosing web-based dispensing during follow-up appointments, the investigator collected the self-reported data on TAI, mMRC, CAT, and COPD-Q via WeChat using a web-based questionnaire or by telephone. In addition, family members of patients with COPD had the option to record a video of the inhaler technique and send it to the investigator via WeChat. The frequency of acute exacerbations and pulmonary function were only assessed at T0 and T7. The SUS scale only measured the satisfaction of patients with COPD in the experimental group at T1. The user activity data could be extracted from the log data of the website.

### Statistical Analyses

All data were entered into Excel 2021 (Microsoft Corp). Following thorough inspection and validation, the database was established, and statistical analysis was conducted by SPSS (version 26.0; IBM SPSS Data Collection). The normality of the data was assessed using the Shapiro-Wilk test. Continuous variables with normal distribution were described as mean (SD), continuous variables with skewed distribution were described as median (IQR), and categorical variables were expressed as frequency and percentage. The demographic and clinical characteristics of both groups were examined using the chi-square test or Fisher exact test (if applicable). Group comparisons of 2 outcome measures were performed by 2 independent samples *t* test or Mann-Whitney U test, and within-group comparisons by paired-sample *t* test or Wilcoxon signed rank test. Generalized estimating equations were used to analyze the changing trend of each outcome indicator at different time points, considering sample size shedding during follow-up. The working correlation matrix was set as exchangeable, and the model was fitted using linear regression to assess time, between-group differences, and their interaction. Effect sizes and pairwise comparisons were conducted using the Bonferroni method. To control the interference from the pretest on intervention effects, the outcome measure at T0 was entered into the equation as a covariate. A 2-sided test with *P*<.05 was used to determine statistically significant differences.

## Results

### General Characteristics of the Sample

**Figure 3:**
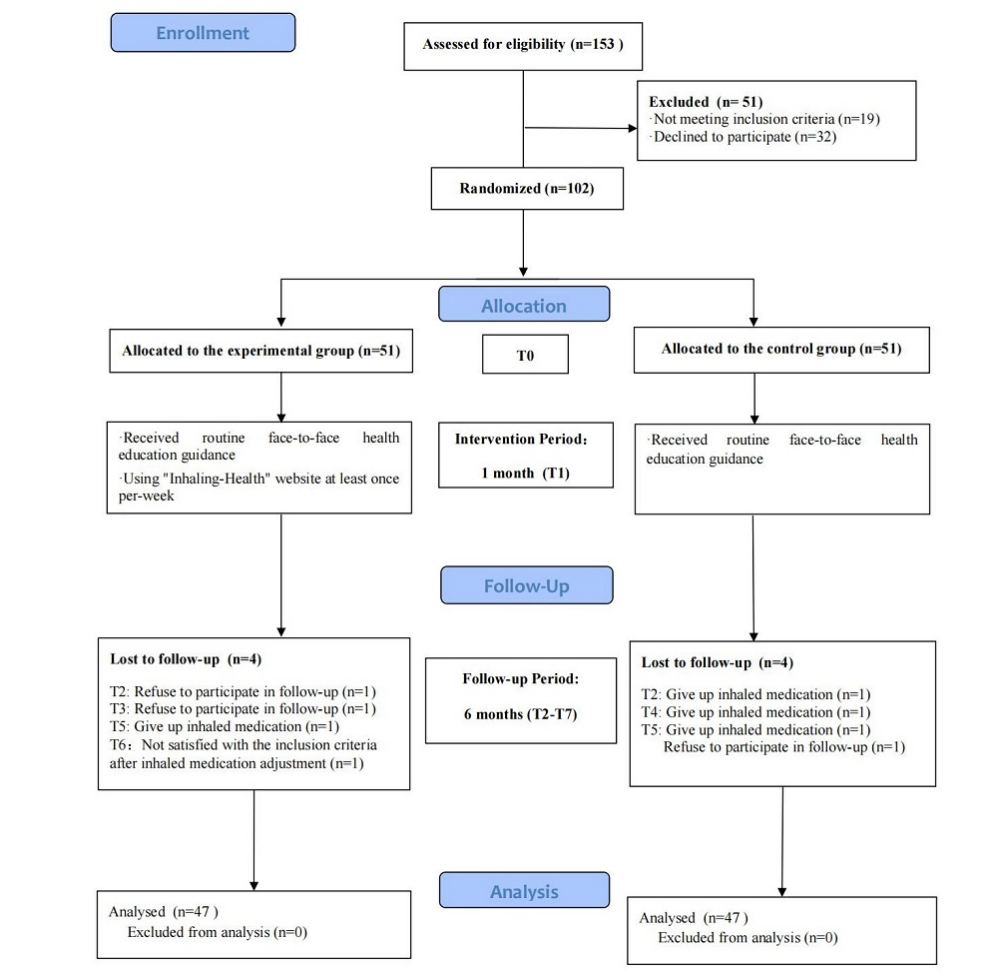
Study flowchart.

A total of 121 patients met the criteria, and 102 (80.3%) patients provided informed consent. They were recruited between April 2023 and May 2023, with 51 patients assigned to the control and experimental group each. Of the 102 patients, 41 (40.2%) were recruited from the First Affiliated Hospital of Zhejiang University School of Medicine and the remaining 61 (59.8%) were recruited from the Hangzhou Normal University Affiliated Hospital. All 102 patients with COPD completed the intervention before July 2023, and 94 (92.2%) patients completed the 6-month follow-up before January 2024. Those lost during the follow-up period were not included in the statistical analysis. A follow-up interruption within 6 months was regarded as an automatic termination of the study. The remaining patients concluded the study after completing the 6-month follow-up (Figure 3). No adverse events occurred during the study.

Among the sample of 102 participants, the majority were male (n=77, 75.5%), with a mean age of 63.48 (SD 11.67) years. Most patients with COPD were married (93/102, 91.2%), had education up to junior high school (73/102, 71.6%), and more than half (59/102, 57.8%) of the patients with COPD had a personal monthly income of >5000 yuan (US $686.5).

In terms of disease and inhalation treatment data, nearly half (52/102, 51%) of the patients with COPD had been diagnosed within the past 5 years. The severity of COPD was moderate to severe, such as GOLD Ⅱ (34/102, 33.3%) and GOLD Ⅲ (32/102, 31.4%). More than half (67/102, 65.7%) of the participants had a history of smoking, as well as other chronic diseases (61/102, 59.8%). On average, the recruited participants used 1.05 (SD 0.22) inhalers, with nearly half (54/102, 52.9%) starting to use inhalers within the last year. Among inhaler types, the pressurized metered-dose inhaler was predominantly used (47/102, 46.1%). The preferred drug prescription was long-acting muscarinic antagonist, long-acting beta-agonist, and inhaled corticosteroid, chosen by most (53/102, 52%) participants.

Appropriate statistical methods were selected for the 2 groups based on variables. The chi-square test was used for categorical variables, the 2 independent samples 2-tailed *t* test was used for normally distributed continuous variables, and the Mann-Whitney U test was used for nonnormally distributed variables. The results indicated that there were no statistically significant differences between the experimental and control groups in terms of demographics, sociological characteristics, diseases, and inhaled medication–related characteristics (*P*>.05), indicating that both groups were comparable (refer to Table 1 for details).

**Table 1:** General data of the 2 groups of patients (N=102).

Characteristics		Control group (n=51)	Experimental group (n=51)	χ^2^ test (*df*)		*t* test (*df*)		*P* value	
**Gender, n (%)**					4.3 (1)		—^a^		.04
	Men	34 (67)	43 (84)						
	Women	17 (33)	8 (16)						
Age (y), mean (SD)		64.65 (12.31)	62.31 (10.99)	—^a^		1.0^b^ (1)		.32	
**Educational level, n (%)**					6.4 (3)		—^a^		.09
	Primary school and below	22 (43)	14 (27)						
	Middle school	20 (39)	17 (33)						
	High school	4 (8)	11 (22)						
	College graduate or beyond	5 (10)	9 (18)						
**Marriage status, n (%)**					—		—		>.99^c^
	Married	46 (90)	47 (92)						
	Single or widowed	5 (10)	4 (8)						
**Living status, n (%)**					0.4 (1)		—		.54
	Live alone	5 (10)	7 (14)						
	Live with others	46 (90)	44 (86)						
**Medical insurance type, n (%)**					—		—^a^		.20^c^
	Without medical insurance	3 (6)	3 (6)						
	New rural cooperative medical care system	13 (25)	6 (12)						
	Basic medical insurance system for urban residents	35 (69)	42 (82)						
**Per capita monthly income (yuan; 1 yuan=US $ 0.14), n (%)**					1.0 (1)		—^a^		.32
	≤5000	32 (63)	27 (53)						
	>5000	19 (34)	24 (47)						
**Duration of COPD diagnosis^d^ (y), n (%)**					—		—^a^		.68^c^
	<5	24 (47)	28 (55)						
	5-10	12 (23)	12 (23)						
	11-15	10 (20)	9 (18)						
	>15	5 (10)	2 (4)						
**GOLD^e^ grade, n (%)**					0.8 (3)		—^a^		.85
	Ⅰ (mild)	10 (20)	12 (23)						
	Ⅱ (moderate)	16 (31)	18 (35)						
	Ⅲ (severe)	18 (35)	14 (27)						
	Ⅳ (very severe)	7 (14)	7 (14)						
**Smoking history, n (%)**					0.4 (2)		—^a^		.79
	Never smoker	10 (20)	12 (23)						
	Former smoker	22 (43)	23 (45)						
	Current smoker	19 (37)	16 (31)						
**Comorbidities status, n (%)**					2.0 (1)		—^a^		.16
	With comorbidities	27 (53)	34 (67)						
	No comorbidities	24 (47)	17 (33)						
Number of comorbidities, mean (SD)		0.75 (0.84)	0.82 (0.71)	—^a^		–0.5^b^ (1)		.61	
**Inhalation duration (y), n (%)**					—		—^a^		.42^c^
	<1	25 (49)	29 (57)						
	1-5	11 (22)	11 (22)						
	6-10	10 (20)	10 (20)						
	>10	5 (10)	1 (2)						
Number of inhaler devices used at the same time, mean (SD)		1.02 (0.14)	1.08 (0.27)	—^a^		–1.4^b^ (74)		.17	
**Inhaler devices type, n (%)**					—		—^a^		.48^c^
	pMDI^f^	25 (49)	22 (43)						
	Handihaler	4 (8)	6 (12)						
	Turbuhaler	9 (18)	4 (8)						
	Accuhaler	3 (6)	4 (8)						
	Breezehaler	9 (18)	11 (22)						
	Used in combination	1 (2)	4 (8)						
**Pharmacotherapy, n (%)**					1.4 (3)		—^a^		.71
	LAMA^g^	4 (8)	6 (12)						
	LABA^h^+ICS^i^	12 (23)	8 (16)						
	LABA+LAMA	9 (18)	11 (22)						
	LABA+LAMA+ICS	26 (51)	26 (51)						

^a^Not applicable.

^b^Independent samples *t* test.

^c^Fisher exact test.

^d^COPD: chronic obstructive pulmonary disease.

^e^GOLD: Global Initiative for Chronic Obstructive Lung Disease.

^f^pMDI: pressurized metered-dose inhaler.

^g^LAMA: long-acting muscarinic antagonist.

^h^LABA: long-acting beta-agonist.

^i^ICS: inhaled corticosteroid.

### Effects of the Intervention: Between-Group Comparison

Table 2 demonstrates that in the initial analysis, the experimental group using the Inhaling-Health website showed a significant advantage over standard care. In particular, there was a significant difference in inhaler technique (*Z*=–4.489; *P*<.001) and health literacy (*Z*=–2.551; *P*=.01) of patients with COPD in the experimental group compared to the control group at T1. However, there were no statistically significant changes between groups in inhalation medication compliance (*Z*=–1.342; *P*=.18), dyspnea (*Z*=–0.254; *P*=.80), and HRQoL (*Z*=–0.513; *P*=.61) at the end of the intervention.

**Table 2:** Comparison of outcome variables between the 2 groups at each time point.

Variable	T0		T1		T2		T3		T4		T5		T6		T7	
	*Z*	*P* value	*Z*	*P* value	*Z*	*P* value	*Z*	*P* value	*Z*	*P* value	*Z*	*P* value	*t* test (*df*)	*P* value	*t* test (*df*)	*P* value
TAI^a^ total score	–0.591	.55	–1.342	.18	–2.014	.04	–3.892	<.001	–3.799	<.001	–4.474	<.001	–5.449 (92)	<.001	–5.685 (92)	<.001
Erratic nonadherent dimension of TAI	–0.191	.85	–0.735	.46	–0.336	.74	–2.344	.02	–2.690	.007	–3.271	.001	–5.289 (92)	<.001	–4.951 (92)	<.001
Deliberate nonadherent dimension of TAI	–1.057	.29	–0.859	.39	–2.001	.045	–4.106	<.001	–3.919	<.001	–2.807	.005	–3.310 (92)	.001	–3.963 (92)	<.001
Accuracy rate of inhaler technique (%)	–0.041	.97	–4.489	<.001	–3.647	<.001	–3.617	<.001	–3.877	<.001	–4.977	<.001	–5.456 (92)	<.001	–5.269 (92)	<.001
Severity of dyspnea (mMRC)^b^	–0.528	.60	–0.254	.80	–0.491	.62	–1.361	.17	–1.880	.06	–2.260	.02	–2.038 (92)	.04	–2.085 (92)	.0^4^
HRQoL^c^ of patients with COPD^d^ (CAT^e^ score)	–1.954	.05	–0.513	.61	1.373	.17	1.678	.10	2.173	.03	3.156	.002	2.939 (92)	.004	3.302 (92)	.002
Health literacy of patients with COPD (COPD-Q^f^ score)	–0.509	.61	–2.551	.01	–2.799	.006	–4.206	<.001	–3.824	<.001	–4.053	<.001	–5.990 (92)	<.001	–6.129 (92)	<.001

^a^TAI: Test of Adherence to Inhalers.

^b^mMRC: modified Medical Research Council.

^c^HRQoL: health-related quality of life.

^d^COPD: chronic obstructive pulmonary disease.

^e^CAT: Chronic Obstructive Pulmonary Disease Assessment Test.

^f^COPD-Q: Chronic Obstructive Pulmonary Disease Knowledge Questionnaire.

**Figure 4:**
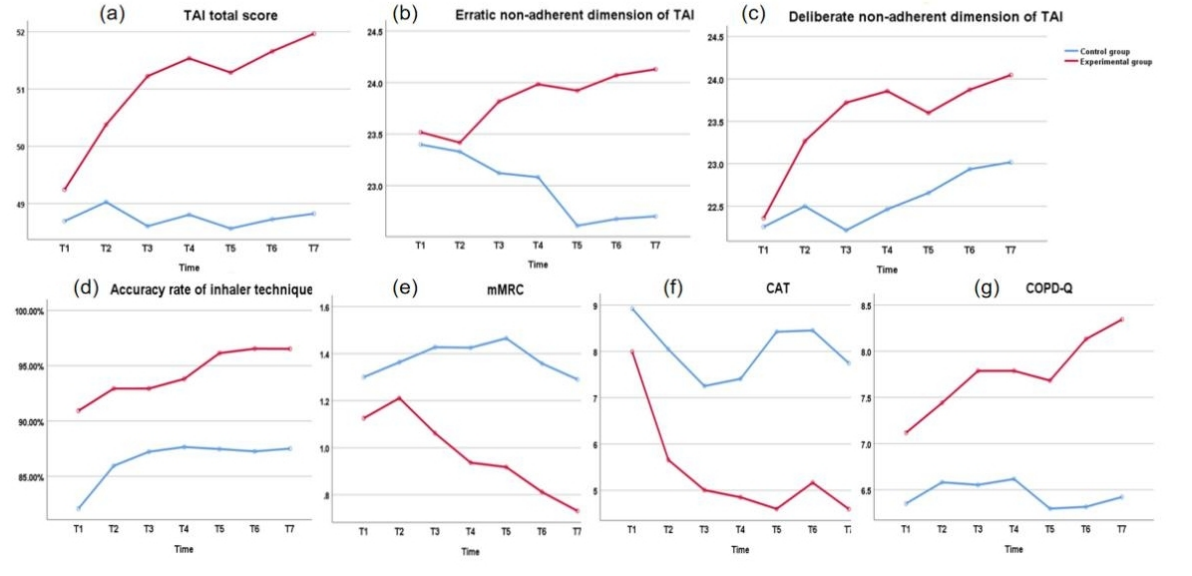
Pretest and posttest means in outcome variables for the experimental and control groups. CAT: Chronic Obstructive Pulmonary Disease Assessment Test; COPD-Q: Chronic Obstructive Pulmonary Disease Knowledge Questionnaire; mMRC: Modified Medical Research Council scale; TAI: Test of Adherence to Inhalers.

Multimedia Appendix 3 and Figure 4 show the differences at the postintervention and 6-month follow-up for the 2 groups. In terms of TAI total score and all dimension scores, inhaler technique accuracy, and COPD-Q score, the scores of the experimental group exhibited a significant upward trend and were consistently higher than those of the control group (95% CI of Wald χ^2^ value at each time point are shown in Multimedia Appendix 3). From T1 to T7, it became increasingly apparent that there was an erratic nonadherent behavioral pattern in the control group, while the statistical difference was evident from T3 to T7. For deliberate nonadherent behavioral patterns, although the experimental group declined at T5, the score of the deliberate dimension of TAI at T7 was significantly higher than that at T1. However, in the control group, a decrease was observed at T3, but the difference between T7 and T1 was limited. The difference between groups remained statistically significant in inhaler technique accuracy and COPD-Q score from T1 to T7. In the control group, the accuracy of the inhaler technique plateaued from T3 to T7, while the experimental group continued to improve after T4. The COPD-Q score of the experimental group decreased from T3 to T5 and then showed a slight increase during the remaining follow-up period. In contrast, the control group exhibited stable COPD-Q scores from T1 to T4, followed by a rapid decline at T5 and subsequent stabilization. In terms of mMRC and CAT scores, the experimental group’s scores decreased overall and were consistently lower than those of the control group. The gap in mMRC scores gradually increased between the 2 groups, but it was only statistically significant from T5 to T7. The trend of CAT scores was similar between T1 and T3, but the difference between the 2 groups gradually increased from T4. The results of the generalized estimating equations analysis of outcome variables are shown in Multimedia Appendix 3.

Furthermore, Table 3 illustrates disparities in pulmonary function and the frequency of acute exacerbation among patients with COPD. The experimental group showed statistically significant improvements in pulmonary function and the frequency of acute exacerbation compared to the baseline (*P*<.05). Intergroup differences at 6 months of follow-up were statistically significant for the frequency of acute exacerbation (*Z*=–2.621; *P*=.009) and FEV1/FVC (*Z*=–1.962; *P*=.05).

**Table 3:** Comparison of acute exacerbation frequency and pulmonary function between the 2 groups of patients with COPD at baseline and after follow-up.

Variable	Control group (n=47)					Experimental group (n=47)					Difference between groups at T7		*P* value
	T0, median (IQR)	T7, median (IQR)	Within-group effect^a^	*P* value	T0, median (IQR)		T7, median (IQR)	Within-group effect	*P* value				
The frequency of acute exacerbation in the recent half-year	0.00 (0.00-1.00)	0.00 (0.00-1.00)	–0.315^b^	.75	1.00 (0.00-1.00)		0.00 (0.00-0.00)	–3.542^b^	<.001	–2.621^c^		.009	
FEV_1_^d^/pre (%)	51.70 (36.29-75.10)	64.50 (39.02-81.70)	–3.238^b^	.001	64.88 (38.78-76.40)		70.50 (44.93-86.52)	–5.206^b^	<.001	–1.887^c^		.06	
FEV_1_ (Liter)	1.32 (0.96-1.85)	1.54 (1.02-2.12)	–2.043^b^	.04	1.46 (1.01-2.09)		1.82 (1.18-2.43)	–4.070^b^	<.001	–1.172^c^		.24	
FVC^e^ (Liter)	2.65 (2.17-3.26)	2.57 (2.19-3.45)	–1.984^b^	.047	2.62 (1.96-3.44)		2.83 (2.19-3.68)	–3.286^b^	<.001	–0.684^c^		.49	
FEV_1_/FVC (%)	53.67 (45.83-63.48)	54.69 (43.93-65.11)	–0.614^b^	.54	60.87 (43.21-67.11)		64.33 (47.47-70.11)	–3.778^b^	<.001	–1.962^c^		.05	

^a^Within-group effect of T7-T0.

^b^Wilcoxon signed rank test.

^c^Mann-Whitney U test.

^d^FEV_1_: forced expiratory volume in the first second.

^e^FVC: forced vital capacity.

### Effects of the Intervention With Pretest Control Covariate

Generalized estimating equation analysis with T0 as a covariate showed no interaction effect on the erratic nonadherent dimension of TAI or the accuracy rate of inhaler technique, indicating that the effect of gamified inhalation medication education program on accidental omission medication behavior and inhaler technique were not affected by time. There were significant interaction effects between group and time for TAI total scores, deliberate nonadherence dimension scores, CAT, COPD-Q, and mMRC scores. Combined with the influence of the time×group effect, the β value of the TAI total score gradually increased. This indicated that the Inhaling-Health website gradually improved the overall inhaled medication compliance of patients with COPD 2 months after the intervention compared to the control group. However, in the erratic nonadherent dimension of TAI, the beta value of the combined effect between time and group reached its highest at T3, which indicated that the website reached its peak effectiveness in correcting intentional nonadherent behavior 2 months after the intervention. In terms of inhaler technique, the Inhaling-Health website had a positive effect on improving accuracy, independent of the effect of time. The mMRC score showed a downward trend since T4, indicating that the Inhaling-Health website was more effective than the routine way of inhaled medication education 3 months following the intervention. As for CAT scores, there was a decrease in the beta value of the time×group effect from T1 to T7. It suggested that the Inhaling-Health website was more effective than the conventional intervention in improving patients’ HRQoL during the follow-up. Regarding patients’ health literacy, as measured by COPD-Q, there was an increasing beta value with the time×group effect, indicating that the Inhaling-Health website could improve patients’ health literacy compared to the routine education method.

### Website Use

**Figure 5:**
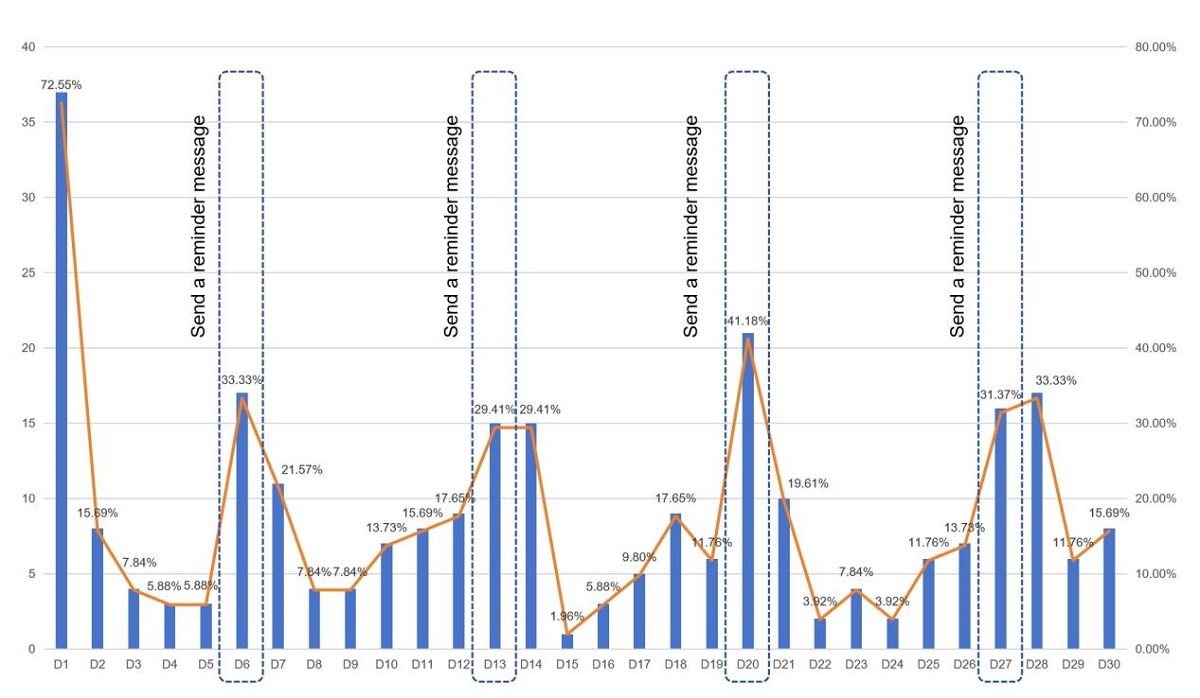
Percentage of daily active users on the Inhaling-Health website (n=51).

Under the weekly guidance of the researchers, all users effectively used the Inhaling-Health website for at least 4 days within the 30-day use period. Among 51 users, the number of active days varied from 4 to 15 days, with a median of 5 (IQR 4-6) days and an average interval between use of 5.50 (SD 1.61) days (refer to Figure 5 for details).

The number of user clicks on the Inhaling-Health website ranged from 5 to 35 times during the intervention period, with a median of 11 (IQR 8-14) times. Effective use and accumulated website points ranged from 15 to 125, with a median total score of 35 (IQR 25-55). The frequency of use in each section of the website and user satisfaction are shown in Table 4. According to the ratio of click frequency and points, the proportion of the Game section was slightly higher than that of the Knowledge Education section. The *Healthy Farmland* game was the most popular part of the Inhaling-Health website. According to the SUS scale evaluation, the average score of 51 users was 82.64 (SD 6.21), indicating that patients with COPD were satisfied with the experience of using the Inhaling-Health website, which helped them manage inhaled medication better.

**Table 4:** User data and user satisfaction of the Inhaling-Health website.

Variable			Mean (SD)		Median (IQR)		Composition ratio (%)
Total clicks on the website			12.49 (6.34)		11.00 (8-14.00)		637 (100)
**Knowledge Education section click frequency**			5.57 (2.68)		5.00 (4.00-6.00)		47.31 (14.63)
	Clicks on educational articles	3.33 (1.95)		3.00 (2.00-4.00)		27.82 (12.32)	
	Clicks on demonstration videos	2.24 (1.18)		2.00 (1.00-3.00)		19.49 (9.64)	
**Game section click frequency**			6.92 (4.71)		6.00 (4.00-9.00)		52.69 (14.63)
	Clicks on the Healthy Skyscraper game	1.76 (1.46)		1.00 (1.00-3.00)		13.34 (9.04)	
	Clicks on the Healthy Farmland game	5.16 (3.65)		4.00 (3.00-6.00)		39.35 (13.38)	
Total points			44.47 (26.15)		35.00 (25.00-75.00)		—^a^
**Points in the Knowledge Education section**			20.27 (13.41)		16.00 (15.00-22.00)		49.09 (16.92)
	Points of educational articles	12.73 (11.57)		10.00 (6.00-15.00)		29.60 (14.52)	
	Points of demonstration videos	7.65 (4.62)		6.00 (5.00-10.00)		19.49 (13.22)	
**Points in the Game section**			24.20 (18.47)		20.00 (10.00-33.00)		50.91 (16.92)
	Points of the Healthy Skyscraper game	8.41 (7.74)		8.00 (0.00-13.00)		16.81 (13.30)	
	Points of the Healthy Farmland game	15.78 (12.42)		15.00 (5.00-20.00)		34.09 (13.92)	
**Total score of SUS^b^**			82.64 (6.21)		82.50 (80.00-87.50)		—
	Dimension of effectiveness^c^	9.02 (1.46)		9.00 (8.00-10.00)		75.16 (12.19)	
	Dimension of usability^d^	13.49 (1.30)		13.00 (13.00-14.00)		84.31 (8.14)	
	Dimension of satisfaction^e^	10.55 (1.08)		11.00 (10.00-11.00)		87.91 (9.02)	

^a^Not applicable.

^b^SUS: system usability scale.

^c^The range of scores for the effectiveness dimension in the SUS scale is 3-12.

^d^The range of scores for the usability dimension in the SUS scale is 4-16.

^e^The range of scores for the satisfaction dimension in the SUS scale is 3-12.

## Discussion

### Principal Findings

This is the first study that applies the Fogg Behavior Model [20] and gamification design elements to enhance the inhalation medication adherence of patients with COPD. It includes an empirical investigation of a 1-month intervention and 6-month follow-up to validate the effectiveness of a gamified inhalation medication education program on compliance with inhaled medication, the accuracy rate of inhaler technique, patients’ symptom severity, pulmonary function, the risk of exacerbations, and health literacy. Strict quality control was implemented throughout the study, and generalized estimating equations analysis was used postintervention to consider covariate and intervention effects on the impact of an educational program on inhalation medication intervention gaming.

At the end of the intervention, there was no significant difference in TAI total scores between groups, indicating that the gamified inhalation medication education program did not have an immediate impact on inhalation medication compliance in patients with COPD. This finding is consistent with the study on medication management in patients with breast cancer by Kim et al [44]. However, further analysis using the TAI questionnaire revealed that the gamified program effectively reduced intentional medication nonadherent behavior in patients with COPD and had an intervention effect on accidental medication reduction behavior. The quiz game on the Inhaling-Health website, along with gamification design elements, such as points, effectively motivated them to actively engage in inhalation medication education, leading to a gradual understanding of treatment importance and timely feedback. As a result, patients with COPD were more likely to develop a habit of regular medication intake in their daily lives.

This study found that the gamified inhalation medication education program had a significant impact on the accuracy of the inhaler technique in patients with COPD. In the experimental group, the median accuracy rate of inhaler technique at the end of the intervention was 90%, which exceeded the results reported in a 6-month health education study by Chinese scholars [45]. Throughout the follow-up period, it was observed that the accuracy of the inhaler technique in patients with COPD in the experimental group was higher than that in the concurrent control group (*P*<.05), indicating that the gamified inhalation medication education program was more effective in helping patients with COPD maintain correct inhalation medication steps compared to conventional methods. This finding was consistent with the gamification-designed mobile app intervention by Wiecek et al [46]. In this study, the Inhaling-Health website transformed complete inhaler technique education into individual game levels, allowing patients with COPD to learn how to use their inhalation device within a gaming context. When a patient made an error at any level, they were prompted to “watch the correct presentation three times.” This approach not only reinforced patients’ memory of proper inhaler use through repeated education but also helped avoid potential embarrassment from medical staff and reduced the psychological burden for patients with COPD [47].

Patients with COPD who participated in a gamified inhalation medication education program demonstrated sustained improvement in health literacy for 6 months following the conclusion of the intervention, with consistently significant differences observed between the 2 groups. This finding may be attributed to the lack of confidence that many patients with COPD have in managing their disease, leading them to assume a more passive role and rely on physician direction for their care [48]. In Inhaling-Health, the joy of successfully answering the question depends on dopamine release [19], which attracts patients to actively and repeatedly learn the knowledge and skills related to inhalation medication. When faced with challenges on the website, patients with COPD were encouraged to seek out information to improve their success rate with subsequent answers. These findings suggested that the Inhaling-Health website effectively harnessed external motivation among patients with COPD to facilitate learning about disease management.

The gamified inhalation education program was shown to be more effective in improving clinical indicators, such as mMRC, CAT, and pulmonary function, compared to conventional inhalation education. In the experimental group of patients with COPD, there was an overall downward trend in mMRC and CAT scores, with a brief rebound observed. This differs from the results of the study by Ceyhan and Tekinsoy Kartin [49], possibly due to the inclusion of breathing exercises in their intervention program for significant improvement in dyspnea symptoms. Given the progressive nature of COPD, a longer follow-up is needed to explain symptom changes. At the end of the follow-up period, this study found significant differences between groups in FEV1/FVC values (*P*=.05), indicating that the gamification inhalation medication education regimen relieved airway obstructive disorders. It contradicts the results obtained by Torres Sánchez et al [50] in a systematic evaluation of video games for obstructive pulmonary disease, which may be attributed to the fact that their study mainly focused on years before 2011 and did not incorporate gamification design concepts into their selected video games, resulting in no significant effect on improving lung function in patients.

### Limitations

The study participants included in this research were limited to patients with COPD in Zhejiang Province, China. Therefore, the conclusions of the study may be geographically restricted. In addition, the follow-up period in this study was <1 year, which ignored the potential confounding effects of seasonal variation on changes in COPD condition. Future multicenter studies are necessary to extend the follow-up time to improve the generalizability of the conclusions. In addition, the use of website could not be measured as an intervention dose. We only extracted log data to describe user activity, demonstrating the website’s effectiveness, usability, and user satisfaction. Future studies will be conducted to clarify the effective intervention dose.

### Conclusions

On the basis of the Fogg Behavior Model [20], this study innovatively integrated gamification into inhalation medication education for patients with COPD combined with game design elements. The web page format not only provides an engaging and user-friendly experience for patients with COPD in learning about inhalation medication but also allows for human-computer interaction, enabling patients to receive timely feedback and improve their compliance with inhalation medication. The empirically validated gamified intervention scheme has strong operability and can be used as a supplementary strategy to enhance the effectiveness of inhalation medication education for patients with COPD. It aims to improve compliance with and accuracy of inhalation medication, enhance health literacy, reduce dyspnea severity, partially improve pulmonary function, decrease the risk of the frequency of acute exacerbations, and comprehensively enhance HRQoL for patients with COPD.

## Appendix

Multimedia Appendix 1 Functions of the Inhaling-Health website.

Multimedia Appendix 2 Questionnaires.

Multimedia Appendix 3 The results of the generalized estimating equations analysis of outcome variables.

Multimedia Appendix 4 CONSORT eHEALTH checklist (V 1.6.1).

## Abbreviations

CAT: Chronic Obstructive Pulmonary Disease Assessment Test
COPD: chronic obstructive pulmonary disease
COPD-Q: Chronic Obstructive Pulmonary Disease Knowledge Questionnaire
FEV1: forced expiratory volume in the first second
FVC: forced vital capacity
GOLD: Global Initiative for Chronic Obstructive Lung Disease
HRQoL: health-related quality of life
mMRC: modified Medical Research Council
SUS: system usability scale
TAI: Test of Adherence to Inhalers

## Data Availability

The datasets generated or analyzed during this study are available from the corresponding author (LH) on reasonable request.
