# Supplementary material for: Effect of Gamification on Improved Adherence to Inhaled Medications in Chronic Obstructive Pulmonary Disease: Randomized Controlled Trial
Source: J Med Internet Res. 2025 May 14;27:e65309. doi: 10.2196/65309 (PMC12120366; doi:10.2196/65309)
Supplement: Multimedia Appendix 1 [file jmir_v27i1e65309_app1.docx]

Multimedia Appendix 1. The function introduction of the "Inhaling-Health" website

Based on Fogg Behavior Model and gamification design principles, combined with the evidence-based basis and panel discussions, we constructed an initial functional framework for the "Inhaling-Health" website (see Figure 1).


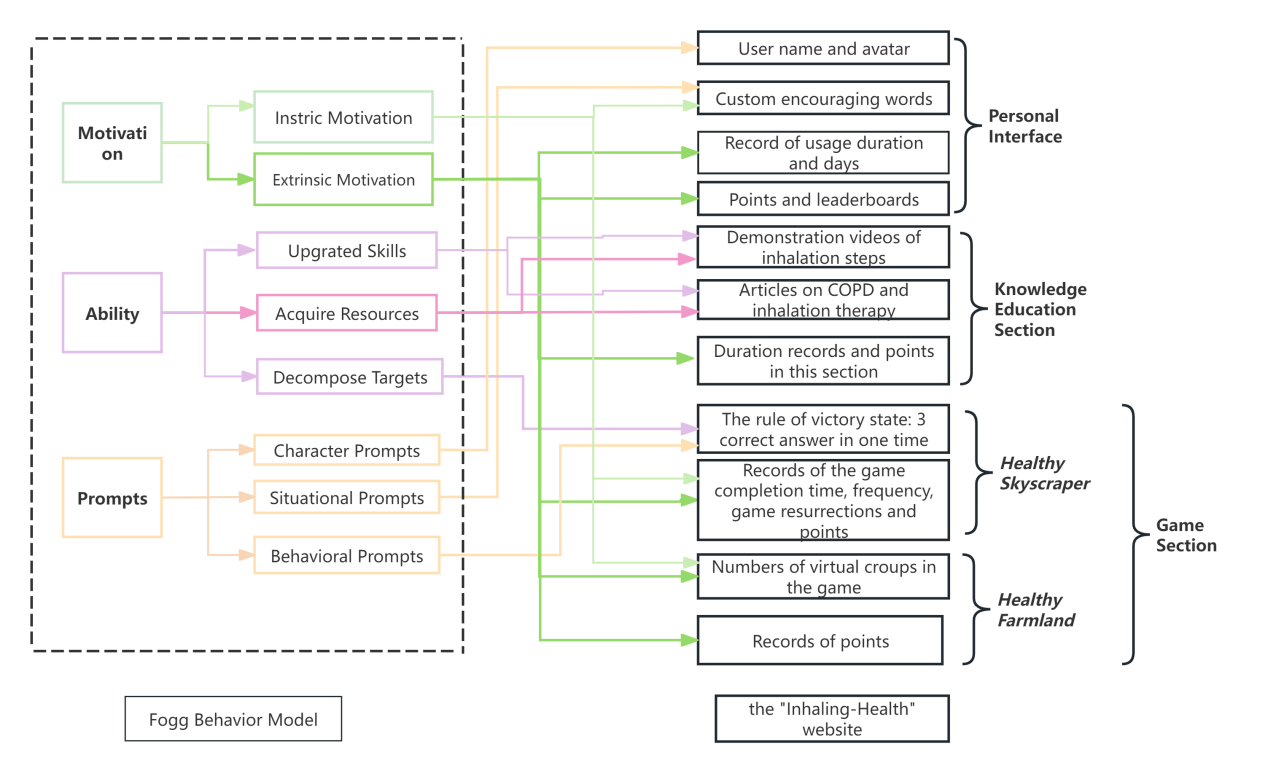


Fig. 1 The functional framework diagram of the "Inhaling-Health" website

User successfully logged in the "Inhaling-Health" website with their personal name and phone number. To more intuitively show users the three functions of the website, personal interface, knowledge and education section and game section, we took the advice of the game designer and designed navigation panel of the "Inhaling-Health" website.

In the personal interface, the user name, user profile, custom motivational words, online duration of the day, number of days using the website and the total points and rankings generated from using the website were recorded. The user name, avatar, and usage days correspond to the character prompts of Fogg Behavior Model, while the "custom motivational discourse" function is the "situational prompt," making the user realize that they are mainly responsible for their health. Allowing patients to see the continuous accumulation of the number of days recorded on this interface gives them the confidence to continue inhalation therapy, which not only intrinsically motivates users to adhere to inhaled medication but also meets the requirements of "allowing users to see timely feedback" in the gamification design principles (See Figure 2).


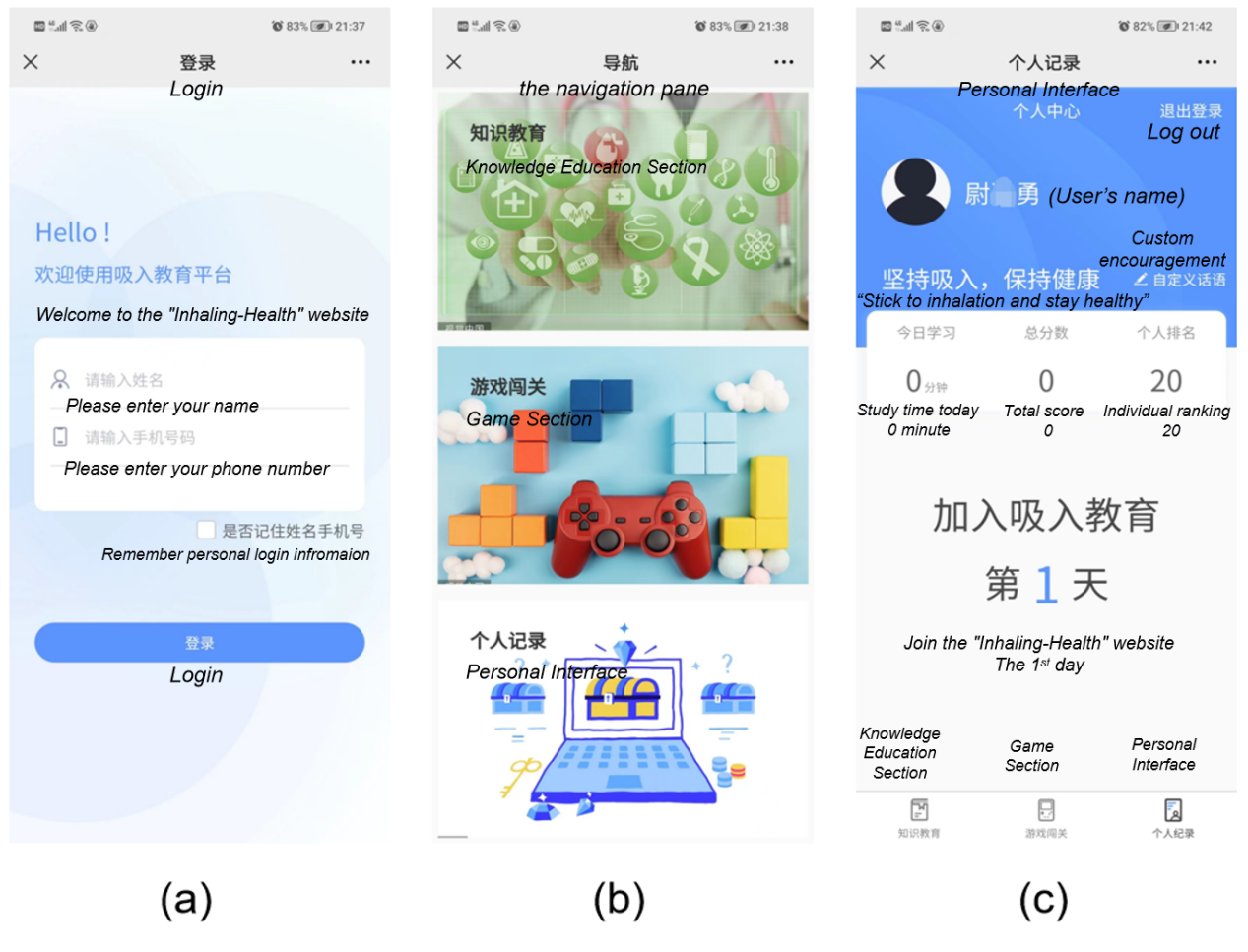


Fig. 2 Screenshot of the personal interface section on the "Inhaling-Health" website. From left to right: (a) the landing interface of the "Inhaling-Health" website, (b) the navigation pane of the "Inhaling-Health" website, (c) the personal interface section.

The Knowledge Education Section includes articles on inhaled medication education and demonstration videos of inhaled medication steps (see Figure 3), which meets the requirements of "improving skills" and "obtaining resources" in the Fogg Behavior Model. COPD patients can enhance their knowledge and ability with multimedia, making it easier to use inhalation devices correctly and persist in long-term inhaled medication behavior. To encourage users to continue using the "Inhaling-Health" website and guarantee the quality control of its intervention, the expert panel determined that it is necessary to define the standards for effective reading of educational articles and effective viewing of educational videos. We standardized that only users who had stayed on the article details page or video playback for more than 30 seconds were considered effective education. Users could earn 5 points per effective education. At the same time, based on the results of the Phase 1 interview, we encouraged communication and encouragement among users. Comments and likes functions were also set up on the detail pages of articles and videos. Users could earn 3 points per comment and 1 point for each like.


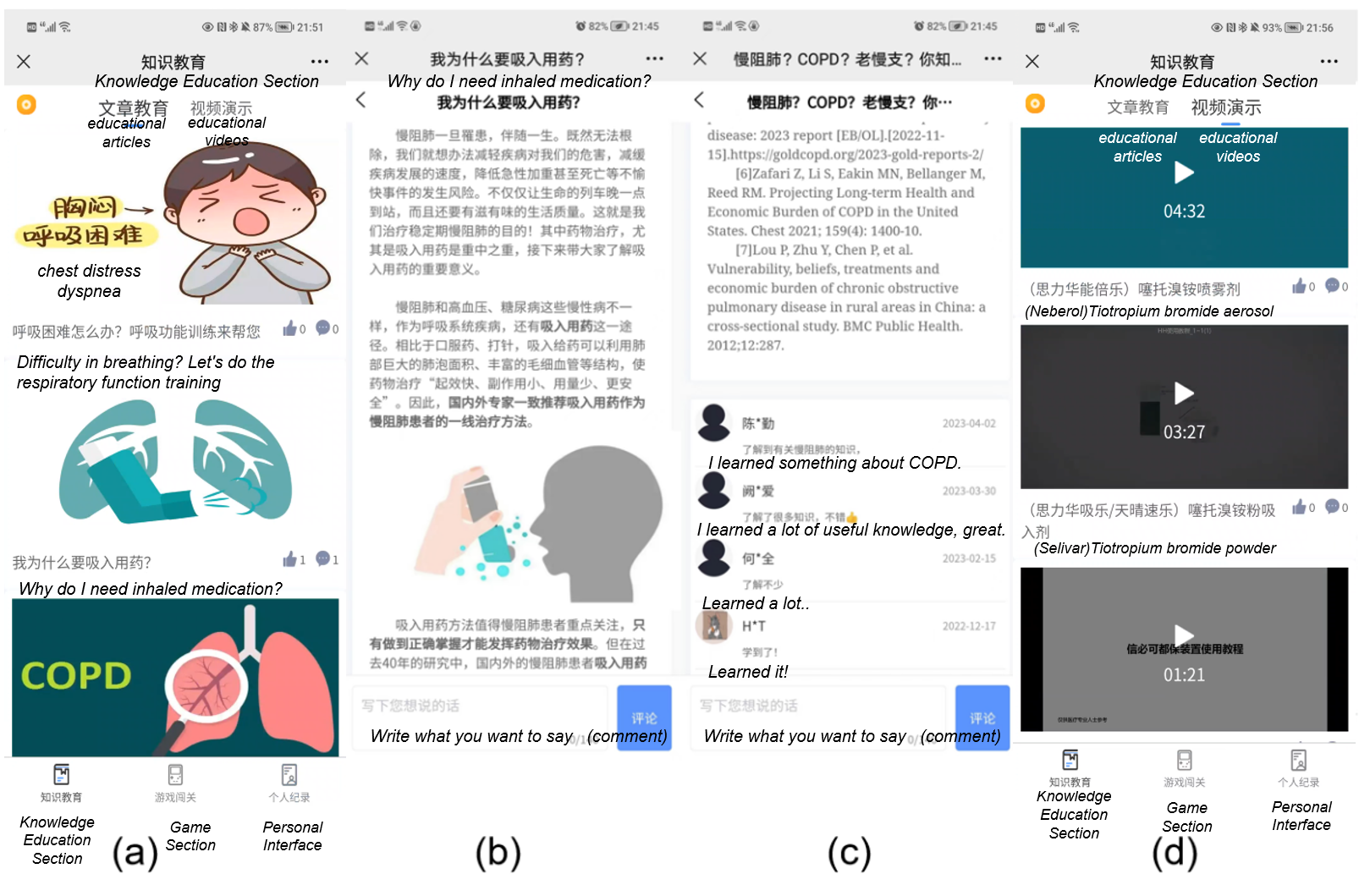


Fig. 3 Screenshot of the Knowledge Education Section on the "Inhaling-Health" website. From left to right: (a) the screen of educational articles, (b) the detailed page of an article, (c) the comment section of the article, (d) the screen of educational videos.

In the Game Section, we have integrated expert opinions and suggestions from participants in Phase 1 to design two games: "*Healthy Skyscraper*" and "*Healthy Farmland*."

In *Healthy Skyscraper*, we referred to the steps for inhaled medication in the Uptodate list and represented these steps as the number of floors required for game characters to climb to the top of the building. This design conformed to Fogg Behavior Model of decomposing movements to make it easier to achieve. Compared to informing or demonstrating the process of inhaled medication to COPD patients at once by the intervention implementer, refining each step into independent levels gives COPD patients pause time for digestion, making each step clear and reducing the difficulty of mastering inhalation techniques. Users who want to pass the game *Healthy Skyscraper* should continuously select the correct option of inhaler application method according to the level prompts. Once fail at any level, users can watch the corresponding short video of the correct operation 3 times for revival, then users continue playing the following levels. The setting of three-time repetition was based on the evidence-based results in Phase 2, which could help COPD patients minimize the error rate of inhalation techniques. There are two ending criteria for the game *Healthy Skyscraper*, either by resurrecting an unlimited number of times until reaching the highest level or by refusing to revive and manually ending the game (See Figure 4).


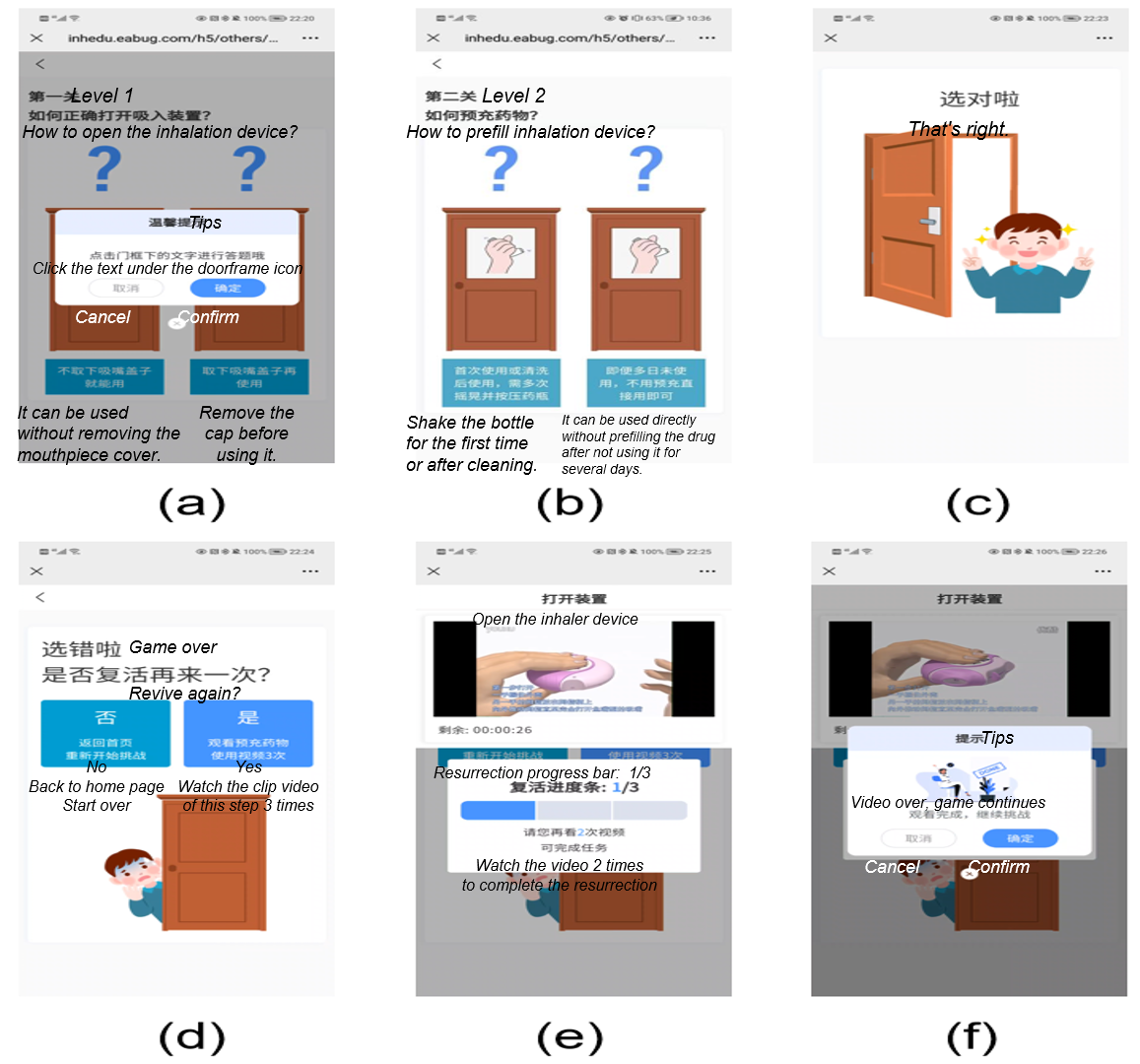


Fig. 4 Screenshot of the game *Healthy Skyscraper* on the "Inhaling-Health" website. From left to right and from top to bottom: (a) the start interface of the game *Healthy Skyscraper*, (b) the game interface, (c) the prompt of the successful level of the game, (d) the option of revive after game failure, (e) the revival requires the player to watch the detailed step video for 3 times (displayed as a progress bar), (f) the interface for successful revival after watching the video.

We considered China's profound agricultural civilization and the preference for planting for the middle-aged and elderly. After integrating the expert group discussion and participants' viewpoints in Phase 1, we combined inhaled medication and disease related knowledge with open-ended question answering challenges to launch the game *Healthy Farmland*. Based on literature and guidelines, we have developed a question bank on self-management and inhaled medication knowledge of COPD. When users choose to plant flowers, the website will randomly select 3 questions from the question bank of inhaled medication knowledge. When users want crops, the website randomly selects 3 questions about disease self-management. Only when the user answers 3 consecutive questions correctly will the estate interface randomly generate corresponding icons as rewards (See Figure 5).


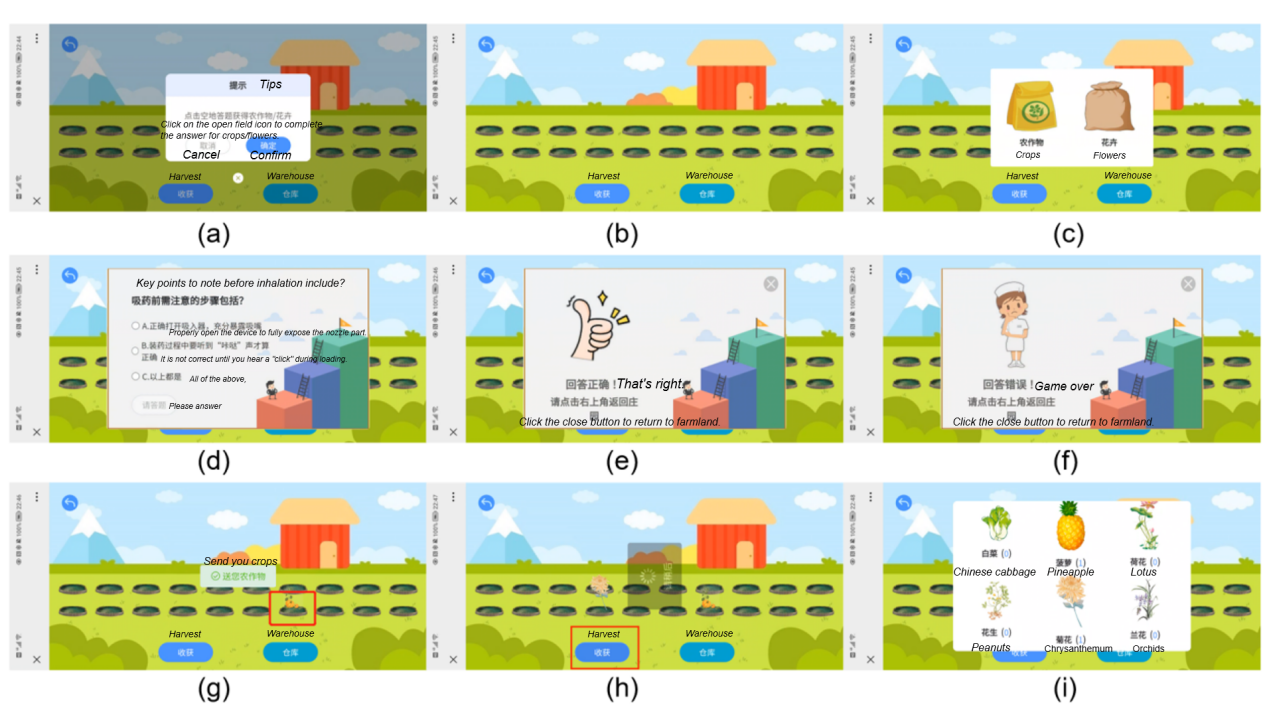


Fig. 5 Screenshot of the game *Healthy Farmland o*n the "Inhaling-Health" website. From left to right and from top to bottom: (a) the start interface of game *Healthy Farmland*, (b) the home page of the game, (c) the option of target crops (corresponding to the question bank of COPD management or medicine inhalation), (d) the game interface, (e) the successful prompt of 3 correct answers of the game, (f) the challenge fail interface for the wrong answer, (g) the main interface shows the random icons after the successful challenge, (h) the jump interface of clicking the "Harvest" button, (i) the detailed page of the warehouse.

After expert group discussion, we also considered that after several rounds of games, the attractiveness to users may decrease. We not only increased the range of in Game Selection by regularly changing the game options of *Healthy Skyscraper* every week but also added the planting category of *Healthy Farmland*, with additional points added for both of the games (see Table 1). Encouraging users to challenge faster and better clearance after becoming familiar with the game also meets their expectations for the puzzle effect that come with the game.

Table 1. Points description of the "Inhaling-Health" website

| Games in the "Inhaling-Health" website | Basic points rule | Cases for extra points |
| --- | --- | --- |
| *Healthy Skyscraper* | 5 points/each pass | ·extra 3 points for no need for revival |
|  |  | ·extra 1 point for the revival times less than 20% of the total levels |
|  |  | ·extra 2 points for finishing the game in less than 5 minutes |
| *Healthy Farmland* | 5 points/each crop or flower | ·extra 3 points for a collection of 10 different flowers |
|  |  | ·extra 3 points for a collection of 10 different crops |
