# Supplementary material for: Effect of Gamification on Improved Adherence to Inhaled Medications in Chronic Obstructive Pulmonary Disease: Randomized Controlled Trial
Source: J Med Internet Res. 2025 May 14;27:e65309. doi: 10.2196/65309 (PMC12120366; doi:10.2196/65309)
Supplement: Multimedia Appendix 2 [file jmir_v27i1e65309_app2.docx]

Multimedia Appendix 2. Questionnaires of the study

1. **General information questionnaire**
2. Demographic information

· Name ______

· Gender

□ Male □ Female

· Age ______ years

· Education level

□ Primary school and below □ Middle school

□ High school □ College graduate and beyond

· Marriage status

□ Married □ Single/ Widowed

· Marriage status

□ Live alone □ Live with others

· Medical payment

□ Without medical insurance □ New rural cooperative medical care system

□ Basic medical insurance system for urban residents

· Per capita monthly income (yuan)

□ ≤5000 □ >5000

1. Disease-related conditions

·Years of diagnosis COPD

□<5 years □5-10 years □11-15 years □>15 years

·Severity of COPD (to be filled in by the investigator after reviewing the most recent pulmonary function test report)

□ [GOLD 1 grade: mild] FEV 1≥80%

□ [GOLD 2 grade: moderate] 50%≤FEV 1<80%

□ [Grade GOLD 3: severe] 30%≤FEV1<50%

□ [Grade GOLD 4: very severe] FEV1<30%

·The frequency of acute exacerbation in the past six months: ________ times

·Smoking history

□ Never smoker □ Former smoker □ Current smoker

· Do you have other chronic diseases?

□ No □ Yes, list:

1. Inhaled medication

·Inhalation duration

□<1 year □1-5 years □6-10 years □>10 years

·The number of inhalation devices used at the same time ______

·Inhaler devices currently used (multiple options):

| 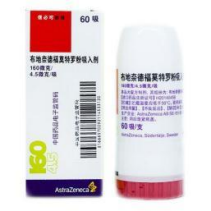  □Budesonide formoterol Powder Inhalant (Symbicor Turbot) | 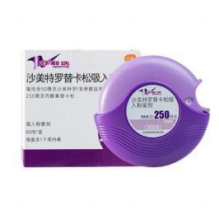  □ Salmeteroticasone Powder inhaler (Sulatide) | 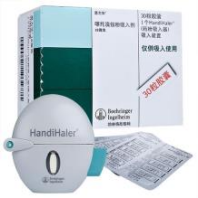  □ Tiotropium bromide powder inhaler (Selivar) |
| --- | --- | --- |
| 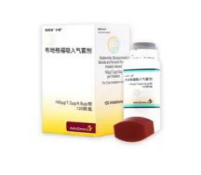  □Budegforo inhalation aerosol (Bezurin) | 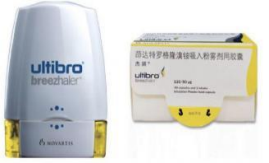  □Indatarrolonium bromide inhaler aerosol (Jerun) | 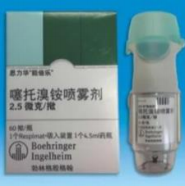  □Tiotropium bromide powder aerosol (Neberol) |

**II. Inhaled Medication Adherence Test (TAI)**

**(Patients domain: questions 1-10)**

1. During the last 7 days, how many times did you forget to take your usual inhalers?

□All □More than half □Approximately a half □Less than half □None

1. Do you forget to take inhalers?

□Always □Mostly □Sometimes □Rarely □Never

1. When you feel good about your illness, do you stop taking your inhalers?

□Always □Mostly □Sometimes □Rarely □Never

1. When you are on vacation or weekend, do you stop taking your inhalers?

□Always □Mostly □Sometimes □Rarely □Never

1. When you are nervous or sad, do you stop taking your inhalers?

□Always □Mostly □Sometimes □Rarely □Never

1. Do you stop taking your inhalers because of fear of side effects?

□Always □Mostly □Sometimes □Rarely □Never

1. Do you stop taking your inhalers because of considering they are useless to treat your condition?

□Always □Mostly □Sometimes □Rarely □Never

1. Do you take fewer inhalations than those prescribed by your doctor?

□Always □Mostly □Sometimes □Rarely □Never

1. Do you stop taking your inhalers because you believe they interfere with your everyday or working life?

□Always □Mostly □Sometimes □Rarely □Never

1. Do you stop taking your inhalers because you have difficulties to pay them?

□Always □Mostly □Sometimes □Rarely □Never

**(Health care professional domain: questions 11-12)**

1. Does the patient remember the prescribed regimen (dose and frequency)? (checking the medical record)

□No □Yes

1. The technique of using the evaluated inhaler device by the patient is (checking the inhalation technique)

□With critical mistakes. □Without critical mistakes.

**Ⅲ. Correctness of inhaler technique (evaluated by the investigator)**

① Sit up ② Open the inhaler device ③ Prepare a dose of drug ④ Exhale

⑤ Keep in the mouth ⑥ Inhale ⑦ Hold your breath ⑧Repeat

⑨ Clean ⑩ Rinse the mouth

Inhaler technique score:

**Ⅳ. The Modified Medical Research Council scale (mMRC)**

□Grade 1 “I only get breathless with strenuous exercise”

□Grade 2 “I get short of breath when hurrying on the level or up a slight hill”

□Grade 3 “I walk slower than people of the same age on the level because of breathlessness or have to stop for breath when walking at my own pace on the level”□Grade 4 “I stop for breath after walking 100 yards or after a few minutes on the level”

□Grade 5 “I am too breathless to leave the house”

**Ⅴ. The COPD Assessment Test (CAT)**

| Symptoms (mild or absent) | Score | Symptoms (severe) |
| --- | --- | --- |
| I never cough. | 0, 1, 2, 3 ,4, 5 | I've been coughing day and night. |
| I don't have any sputum. | 0, 1, 2, 3 ,4, 5 | I have a lot of phlegm. |
| I don't feel any chest distress. | 0, 1, 2, 3 ,4, 5 | I have a terrible feeling of chest distress. |
| When I climb a hill or go up a flight of stairs, I don't feel breathless. | 0, 1, 2, 3 ,4, 5 | When I was climbing or on a floor, I feel very overwhelmed. |
| I can do anything at home. | 0, 1, 2, 3 ,4, 5 | I do anything in the home is affected. |
| Despite my lung disease, I feel confident about going away from home. | 0, 1, 2, 3 ,4, 5 | Because of my lung disease, I don't feel confident about leaving home at all. |
| My sleep quality is good. | 0, 1, 2, 3 ,4, 5 | Because of my lung disease, my sleep quality is quite poor. |
| I am an energetic. | 0, 1, 2, 3 ,4, 5 | I have no energy at all. |

**Ⅵ. The Chronic Obstructive Pulmonary Disease Knowledge Questionnaire (COPD-Q)**

1. COPD is preventable.

□ Yes □No □Unknown

1. COPD is reversible.

□ Yes □No □Unknown

1. People with COPD should be vaccinated against pneumonia.

□ Yes □No □Unknown

1. People with COPD should get an annual influenza vaccine.

□ Yes □No □Unknown

1. Long-term oxygen therapy can prolong the life of COPD patients

□ Yes □No □Unknown

1. Medicine can stop COPD from getting worse.

□ Yes □No □Unknown

1. The symptoms were relieved, and the COPD patients could voluntarily stop using long-acting inhaled drugs.

□ Yes □No □Unknown

1. COPD patients have a persistent cough.

□ Yes □No □Unknown

1. Patients with COPD have symptoms of shortness of breath.

□ Yes □No □Unknown

1. Quitting smoking can prevent COPD from getting worse.

□ Yes □No □Unknown

1. The majority of COPD is caused by smoking or inhaling second-hand smoke.

□ Yes □No □Unknown

1. Salbutamol (a hormone drug) or your usual inhaled medication can be used at any time of shortness of breath.

□ Yes □No □Unknown

1. When breathing difficulties, chronic obstructive pulmonary disease people should only use of inhaled drugs.

□ Yes □No □Unknown

**Ⅶ. The System Usability Scale (SUS)**

| **No.** | **Items** | **Strongly disagree(1) → Strongly agree(5)** | | | | |
| --- | --- | --- | --- | --- | --- | --- |
| 1 | I am willing to use this system. |  |  |  |  |  |
| 2 | I find that using this system is too complex. |  |  |  |  |  |
| 3 | I think the system is easy to use. |  |  |  |  |  |
| 4 | I think I need professional help to use the system. |  |  |  |  |  |
| 5 | I find that the functions in the system fit well together. |  |  |  |  |  |
| 6 | I think there are a lot of inconsistencies in the system. |  |  |  |  |  |
| 7 | I can imagine most people learning to use this system quickly. |  |  |  |  |  |
| 8 | I think this system is very complicated to use. |  |  |  |  |  |
| 9 | I am very confident when using this system. |  |  |  |  |  |
| 10 | I need to learn a lot before I can use this system. |  |  |  |  |  |
