# Supplementary material for: Effect of Gamification on Improved Adherence to Inhaled Medications in Chronic Obstructive Pulmonary Disease: Randomized Controlled Trial
Source: J Med Internet Res. 2025 May 14;27:e65309. doi: 10.2196/65309 (PMC12120366; doi:10.2196/65309)
Supplement: Multimedia Appendix 3 [file jmir_v27i1e65309_app3.docx]

**Table.** The results of the generalized estimating equations analysis of outcome variables.

| Variable | | T1 | | | T2 | | | T3 | | | | | | T4 | | | | T5 | | | | | | | T6 | | | | | T7 | | | |
| --- | --- | --- | --- | --- | --- | --- | --- | --- | --- | --- | --- | --- | --- | --- | --- | --- | --- | --- | --- | --- | --- | --- | --- | --- | --- | --- | --- | --- | --- | --- | --- | --- | --- |
|  |  | β | Wald *χ*^2^ | Wald *χ*^2^ 95% CI | β | Wald *χ*^2^ | Wald *χ*^2^ 95% CI | β | | Wald *χ*^2^ | | | Wald *χ*^2^ 95% CI | β | Wald *χ*^2^ | Wald *χ*^2^ 95% CI | | β | Wald *χ*^2^ | | | Wald *χ*^2^ 95% CI | | | β | Wald *χ*^2^ | | | Wald *χ*^2^ 95% CI | β | | Wald *χ*^2^ | Wald *χ*^2^ 95% CI |
|  | | | | | | | | | | | | | | | | | | | | | | | | | | | | | | | | | |
| **TAI^a^ total score** | | | | | | | | | | | | | | | | | | | | | | | | | | | | | | | | | |
|  | | | | | | | | | | | | | | | | | | | | | | | | | | | | | | | | | |
|  | Time effect | 0^b^ | — | — | 0.515 | 2.4 | −0.133 to 1.162 | −0.045 | | 0.01 | | | −0.953 to 0.862 | 0.202 | 0.2 | −0.782 to 1.185 | | 0.054 | 0.007 | | | −1.198 to 1.305 | | | 0.224 | 0.2 | | | −0.850 to 1.297 | 0.330 | | 0.3 | −0.785 to 1.446 |
|  | Time×group effect | 0^c^ | — | — | 0.557 | 1.2 | −0.445 to 1.599 | 1.924 | | 9.0 | | | 0.664 to 3.183 | 1.942 | 8.0 | 0.596 to 3.288 | | 2.033 | 4.9 | | | 0.235 to 3.832 | | | 2.142 | 7.4 | | | 0.596 to 3.688 | 2.334 | | 8.0 | 0.715 to 3.952 |
|  | | | | | | | | | | | | | | | | | | | | | | | | | | | | | | | | | |
| **Erratic nonadherent dimension of TAI** | | | | | | | | | | | | | | | | | | | | | | | | | | | | | | | | | |
|  |  | | | | | | | | | | | | | | | | | | | | | | | | | | | | | | | | |
|  | Time effect | 0^b^ | — | — | −0.069 | 0.1 | −0.424 to 0.285 | −0.03 | | 0.02 | | | −0.431 to 0.373 | 0.046 | 0.04 | −0.386 to 0.478 | | −0.118 | 0.2 | | | −0.673 to 0.436 | | | −0.041 | 0.03 | | | −0.530 to 0.447 | 0.001 | | <0.001 | −0.508 to 0.510 |
|  | Time×group effect^d^ | — | — | — | — | — | — | — | | — | | | — | — | — | — | | — | — | | | — | | | — | — | | | — | — | | — | — |
|  | | | | | | | | | | | | | | | | | | | | | | | | | | | | | | | | | |
| **Deliberate nonadherent dimension of TAI** | | | | | | | | | | | | | | | | | | | | | | | | | | | | | | | | | |
|  |  | | | | | | | | | | | | | | | | | | | | | | | | | | | | | | | | |
|  | Time effect | 0^b^ | — | — | 0.411 | 5.2 | 0.058 to 0.763 | 0.071 | | 0.09 | | | −0.388 to 0.529 | 0.338 | 1.8 | −0.157 to 0.833 | | 0.597 | 3.5 | | | −0.028 to 1.222 | | | 0.895 | 8.4 | | | 0.291 to 1.499 | 0.980 | | 10.0 | 0.371 to 1.589 |
|  | Time×group effect | 0^c^ | — | — | 0.461 | 2.8 | −0.075 to 0.998 | 1.182 | | 14.0 | | | 0.562 to 1.802 | 1.077 | 9.7 | 0.401 to 1.754 | | 0.575 | 1.7 | | | −0.299 to 1.448 | | | 0.506 | 1.6 | | | −0.273 to 1.285 | 0.591 | | 2.1 | −0.217 to 1.399 |
|  | | | | | | | | | | | | | | | | | | | | | | | | | | | | | | | | | |
| **Accuracy rate of inhaler technique (%)** | | | | | | | | | | | | | | | | | | | | | | | | | | | | | | | | | |
|  |  | | | | | | | | | | | | | | | | | | | | | | | | | | | | | | | | |
|  | Time effect | 0^b^ | — | — | 2.973 | 13.1 | 1.365 to 4.582 | 3.597 | | 19.5 | | | 2.002 to 5.193 | 4.209 | 26.8 | 2.614 to 5.803 | | 5.279 | 43.1 | | | 3.704 to 6.855 | | 5.437 | | 38.6 | | | 3.721 to7.153 | 5.554 | | 44.0 | 3.913 to 7.195 |
|  | Time×group effect | — | — | — | — | — | — | — | | — | | | — | — | — | — | | — | — | | | — | | — | | — | | | — | — | | — | — |
|  | | | | | | | | | | | | | | | | | | | | | | | | | | | | | | | | | |
| **Severity of dyspnea (mMRC^e^)** | | | | | | | | | | | | | | | | | | | | | | | | | | | | | | | | | |
|  |  | | | | | | | | | | | | | | | | | | | | | | | | | | | | | | | | |
|  | Time effect | 0^b^ | — | — | 0.080 | 2.1 | −0.029 to 0.189 | | 0.120 | | 1.9 | −0.052 to 0.292 | | 0.135 | 2.5 | | −0.033 to 0.304 | 0.173 | | 3.7 | −0.004 to 0.351 | | 0.067 | | | | 0.5 | −0.121 to 0.255 | | | 0.003 | 0.001 | −0.182 to 0.188 |
|  | Time×group effect | 0^c^ | — | — | −0.043 | 0.2 | −0.256 to 0.169 | | −0.246 | | 3.4 | −0.509 to 0.017 | | −0.384 | 6.8 | | −0.672 to −0.095 | −0.449 | | 9.0 | −0.743 to −0.155 | | −0.429 | | | | 7.7 | −0.732 to −0.127 | | | −0.451 | 8.2 | −0.760 to −0.142 |
|  | | | | | | | | | | | | | | | | | | | | | | | | | | | | | | | | | |
| **HRQoL^f^ of patients with COPD^g^ (CAT^h^ score)** | | | | | | | | | | | | | | | | | | | | | | | | | | | | | | | | | |
|  | Time effect | 0^b^ | — | — | −0.792 | 3.4 | −1.634 to 0.050 | | −1.512 | | 15.8 | −2.258 to −0.766 | | −1.229 | 7.5 | | −2.108 to −0.351 | −0.237 | | 0.2 | −1.378 to 0.904 | | −0.130 | | | | 0.05 | −1.315 to 1.054 | | | −0.811 | 2.4 | −1.837 to 0.214 |
|  | Time×group effect | 0^c^ | — | — | −1.663 | 9.3 | −2.734 to −0.592 | | −1.830 | | 8.2 | −3.081 to −0.579 | | −2.296 | 12.4 | | −3.573 to −1.020 | −3.467 | | 17.7 | −5.080 to −1.853 | | −3.111 | | | | 13.3 | −4.780 to −1.442 | | | −3.026 | 14.1 | −4.602 to −1.449 |
|  | | | | | | | | | | | | | | | | | | | | | | | | | | | | | | | | | |
| **Health literacy of patients with COPD (Chronic Obstructive Pulmonary Disease Knowledge Questionnaire score)** | | | | | | | | | | | | | | | | | | | | | | | | | | | | | | | | | |
|  | Time effect | 0^b^ | — | — | 0.228 | 6.1 | 0.046 to 0.409 | | 0.188 | | 2.7 | −0.035 to 0.411 | | 0.266 | 4.7 | 0.026 to 0.506 | | −0.047 | | 0.1 | −0.302 to 0.208 | | −0.026 | | | | 0.04 | −0.277 to 0.225 | | | 0.080 | 0.3 | −0.217 to 0.377 |
|  | Time×group effect | 0^c^ | — | — | 0.089 | 0.4 | −0.177 to 0.356 | | 0.463 | | 7.2 | 0.124 to 0.801 | | 0.365 | 4.1 | | 0.010 to 0.719 | 0.573 | | 6.8 | 0.141 to 1.004 | | 1.017 | | | | 24.8 | 0.616 to 1.417 | | | 1.123 | 22.8 | 0.662 to 1.584 |

^a^TAI: Test of Adherence to Inhalers.

^b^T0 as reference.

^c^Control group as reference.

^d^Model effect tests showed no statistically significant difference in time×group effect.

^e^mMRC: Modified Medical Research Council scale.

^f^HRQoL: health-related quality of life.

^g^COPD: chronic obstructive pulmonary disease.

^h^CAT: Chronic Obstructive Pulmonary Disease Assessment Test
